# Supplementary material for: A consolidated and geolocated facility list in Senegal from triangulating secondary data
Source: Sci Data. 2024 Jan 24;11:119. doi: 10.1038/s41597-024-02968-z (PMC10808422; doi:10.1038/s41597-024-02968-z)
Supplement: Supplementary file 1 — Supplementary information [file 41597_2024_2968_MOESM1_ESM.pdf]

Supplementary information

Table of contents	Pages
Table S1. Total number of health facilities, by type and with geolocation, in Senegal and by region.	S2-3
Table S2. Variable descriptions for the full facility list in Senegal.	S4-5
Table S3. Comparing frequency of multiple facility types and facility ownership categorizations, by facility type, in Senegal and by region.	S6-7
Table S4. Comparing GPS availability and concordance, by facility type, in Senegal and by region.	S8-9
Table S5. Comparing number of facilities in the consolidated facility list with facility numbers reported in previously published facility lists by facility type, by region.	S10-11
Table S6. Comparing number of facilities with GPS in the consolidated facility list with facility numbers from Maina and colleagues, nationally and by region.	S12
Supplementary file 1. Report from the facility list workshop in Dakar, Senegal from January 31-February 1, 2023 (available in French only).	S13-20

Table S1. Total number of health facilities, by type and with geolocation, in Senegal and by region. All facilities here reflect hospitals, health centers, health posts, and health huts (i.e., not facilities designated as "other").

Facility type	Location	Total facilities	Total facilities with GPS	Percentage of facilities with GPS (%)
**All facilities**	**Senegal**	**4,685**	**2,423**	**51.7%**
	Dakar	797	328	41.2%
	Diourbel	173	151	87.3%
	Fatick	296	158	53.4%
	Kaffrine	268	126	47.0%
	Kaolack	370	218	58.9%
	Kedougou	139	81	58.3%
	Kolda	327	130	39.8%
	Louga	582	191	32.8%
	Matam	163	128	78.5%
	Saint-Louis	265	204	77.0%
	Sedhiou	204	102	50.0%
	Tambacounda	317	161	50.8%
	Thies	524	261	49.8%
	Ziguinchor	260	184	70.8%
**Hospitals**	**Senegal**	**81**	**70**	**86.4%**
	Dakar	35	30	85.7%
	Diourbel	6	6	100%
	Fatick	2	2	100%
	Kaffrine	2	2	100%
	Kaolack	4	1	25.0%
	Kedougou	1	1	100%
	Kolda	2	2	100%
	Louga	3	2	66.7%
	Matam	3	3	100%
	Saint-Louis	5	5	100%
	Sedhiou	3	2	66.7%
	Tambacounda	1	1	100%
	Thies	12	11	91.7%
	Ziguinchor	2	2	100%
**Health centers**	**Senegal**	**257**	**227**	**88.3%**
	Dakar	82	77	93.9%
	Diourbel	11	8	72.7%
	Fatick	9	9	100%
	Kaffrine	6	4	66.7%
	Kaolack	43	42	97.7%
	Kedougou	5	5	100%
	Kolda	9	7	77.8%
	Louga	30	17	56.7%
	Matam	5	5	100%
	Saint-Louis	12	11	91.7%
	Sedhiou	7	5	71.4%

Facility type	Location	Total facilities	Total facilities with GPS	Percentage of facilities with GPS (%)
	Tambacounda	12	12	100%
	Thies	16	15	93.8%
	Ziguinchor	10	10	100%
**Health posts**	**Senegal**	**2,349**	**1,596**	**67.9%**
	Dakar	680	221	32.5%
	Diourbel	123	109	88.6%
	Fatick	132	108	81.8%
	Kaffrine	105	85	81.0%
	Kaolack	151	132	87.4%
	Kedougou	48	39	81.3%
	Kolda	101	76	75.2%
	Louga	161	126	78.3%
	Matam	101	90	89.1%
	Saint-Louis	136	125	91.9%
	Sedhiou	75	61	81.3%
	Tambacounda	162	118	72.8%
	Thies	228	179	78.5%
	Ziguinchor	146	127	87.0%
**Health huts**	**Senegal**	**1,998**	**530**	**26.5%**
	Dakar	-	-	-
	Diourbel	33	28	84.8%
	Fatick	153	39	25.5%
	Kaffrine	155	35	22.6%
	Kaolack	172	43	25.0%
	Kedougou	85	36	42.4%
	Kolda	215	45	20.9%
	Louga	388	46	11.9%
	Matam	54	30	55.6%
	Saint-Louis	112	63	56.3%
	Sedhiou	119	34	28.6%
	Tambacounda	142	30	21.1%
	Thies	268	56	20.9%
	Ziguinchor	102	45	44.1%

Table S2. Variable descriptions for the full facility list in Senegal. Variable descriptions for the full facility list are available in Table 4 of the manuscript.

**Full facility list dataset**	
File name	senegal_full_facilitylist_full.csv
Total observations	12,965
**Full facility list variables**	
**Variable name**	**Variable description**
region	Region in Senegal.
department	Formal second-level administrative unit for Senegal, with n=45. *Note:* Data sources varied in their reporting of departments or health districts (and did not always specify which was included). Mapping capturing department information and accounting for inaccuracies occurred where possible, but some facility-source observations may not have a department included.
health_district	The peripheral level of Senegal's health sector, with an n=77 to 79. *Note:* Data sources varied in their reporting of departments or health districts (and did not always specify which was included). Mapping from district to department and accounting occurred where possible, but some facility-source observations may not have a health district included.
match_id	Facility id assigned for each unique facility grouping identified in the consolidated facility list. This identifier links facility groups in this dataset to unique observations found in the consolidated facility list. *Note:* this id does not correspond with a particular data source or formal health information system (e.g., DHIS2).
match_name	Processed version of the health facility name used for matching, excluding special characters, variations found across sources.
fac_name_orig	Original health facility name, as found in a given facility data source.
group_fac_type	This is the facility type assigned to a unique facility observation. Original facility types may vary by source, of which are listed under "fac_type_orig." Facility types included in this dataset are as follows in French (without special characters), with English translations: English translations:<br>- hopital= hospital<br>- centre de esante = health center<br>- poste de esante = health post<br>- case de sante= health hut<br>- autre = other
fac_type_orig	Original facility type, as provided in a given facility data source.
group_fac_own	Processed version of the managing authority for a given facility. Types included in this dataset are as follows in French (without special characters), with English translations:<br>- publique = public<br>- prive = private<br>- ong ou mission/confessionnel = NGO or mission/faith-based<br>- paramilitaire = paramilitary
fac_own_orig	Managing authority for the health facility, as provided in a given facility data source.

Full facility list variables	
**Variable name**	**Variable description**
latitude	Latitude coordinates, as provided in a given data source.
longitude	Longitude coordinates, as provided in a given data source.
max_gps_dist	Maximum Haversine distance in kilometers between GPS coordinates for each unique facility grouping. If a facility has only set of GPS coordinates (n_gps==1), this variable is blank.
source	Facility data source.
data_flagged	Variable whereby "1" indicates outstanding verification needs or follow-up questions.
data_notes	Further details about outstanding verification needs or follow-up questions about the given facility. All notes are in English at present. If there is information in "data_notes" but the facility is not flagged (data_flagged==1), these notes are meant to provide additional detail or information about a given facility without immediate action items or follow-up.
decision_notes	Further detail about decisions made for matching facilities and feedback from regional focal points. *All notes are in English at present.*
last_updated	Date of last update in YYYYMMDD format. For instance, 20230518 is 18 May 2023. These updates are meant to reflect changes to previous facility matches since the Dakar facility list workshop that occurred from 31 January to 1 February 2023.
update_notes	Further details on the updates that occurred for a unique facility observation. *All notes are in English at present.*
n_gps	Number of linked sources with GPS for each matched facility group.<br>*Note:* For a given facility, if multiple GPS coordinates were available and matched precisely, they were not counted as distinct sets of GPS.
n_source	Number of linked sources for each matched facility group.
source_list	List of sources for each matched facility group.
fac_id	Unique facility id, either directly provided from its original data source or generated in order to provide a unique identifier for each facility observation.
fac_id_orig	Original facility id, as provided in a given facility data source. *Note*: most non-survey data sources did not have an original facility id, and thus this variable is blank for them.
fac_id_orig_var	Variable name in which facility ids are contained, as found in a given facility data source.<br>*Note:* most non-survey data sources did not have an original facility id, and thus this variable is blank for them.
n_geos	Number of department-health district combinations within a matched facility group. This variable was included to more easily identify which matched facilities need further geographic variation, such that:<br>- If n_geos==0, then no department or health district information were included for these facilities.<br>- If n_geos==1, department-health district combinations for a matched facility group exist and are consistent.<br>- If n_geos >1, department-health district combinations vary within a matched facility group and further verification is needed.

Table S3. Comparing frequency of multiple facility types and facility ownership categorizations, by facility type, in Senegal and by region. All facilities here reflect hospitals, health centers, health posts, and health huts. All facilities here reflect hospitals, health centers, health posts, and health huts (i.e., not facilities designated as "other").

Facility type	Location	Total facilities	Total facilities with >1 facility ownership (% of total facilities)	Total facilities originally with >1 facility type (% of total facilities)	Total facilities originally with >1 facility type and then triangulated or verified (% of total facilities with > 1 facility type)
**All facilities**	**Senegal**	**4,685**	1,895 (40.4%)	486 (10.4%)	202 (41.6%)
	Dakar	797	232 (29.1%)	95 (11.9%)	48 (50.5%)
	Diourbel	173	108 (62.4%)	19 (11%)	9 (47.4%)
	Fatick	296	127 (42.9%)	31 (10.5%)	17 (54.8%)
	Kaffrine	268	85 (31.7%)	20 (7.5%)	0 (0%)
	Kaolack	370	132 (35.7%)	42 (11.4%)	27 (64.3%)
	Kedougou	139	37 (26.6%)	8 (5.8%)	0 (0%)
	Kolda	327	82 (25.1%)	29 (8.9%)	20 (69.0%)
	Louga	582	239 (41.1%)	34 (5.8%)	5 (14.7%)
	Matam	163	89 (54.6%)	10 (6.1%)	10 (100%)
	Saint-Louis	265	139 (52.5%)	44 (16.6%)	9 (20.5%)
	Sedhiou	204	112 (54.9%)	17 (8.3%)	7 (41.2%)
	Tambacounda	317	198 (62.5%)	82 (25.9%)	20 (24.4%)
	Thies	524	189 (36.1%)	39 (7.4%)	18 (46.2%)
	Ziguinchor	260	126 (48.5%)	16 (6.2%)	12 (75.0%)
**Hospitals**	**Senegal**	**81**	12 (34.3%)	4 (11.4%)	2 (50.0%)
	Dakar	35	3 (50.0%)	1 (16.7%)	0 (0%)
	Diourbel	6	2 (100%)	2 (100%)	1 (50.0%)
	Fatick	2	1 (50.0%)	1 (50.0%)	0 (0%)
	Kaffrine	2	1 (25.0%)	1 (25.0%)	0 (0%)
	Kaolack	4	0 (0%)	0 (0%)	-
	Kedougou	1	2 (100%)	0 (0%)	-
	Kolda	2	0 (0%)	0 (0%)	-
	Louga	3	0 (0%)	0 (0%)	-
	Matam	3	1 (20.0%)	1 (20.0%)	0 (0%)
	Saint-Louis	5	1 (33.3%)	0 (0%)	-
	Sedhiou	3	0 (0%)	0 (0%)	-
	Tambacounda	1	3 (25.0%)	2 (16.7%)	1 (50.0%)
	Thies	12	0 (0%)	1 (50.0%)	0 (0%)
	Ziguinchor	2	12 (34.3%)	4 (11.4%)	2 (50.0%)
**Health centers**	**Senegal**	**257**	147 (57.2%)	70 (27.2%)	47 (67.1%)
	Dakar	82	44 (53.7%)	25 (30.5%)	22 (88.0%)
	Diourbel	11	8 (72.7%)	3 (27.3%)	3 (100%)
	Fatick	9	8 (88.9%)	5 (55.6%)	3 (60.0%)
	Kaffrine	6	5 (83.3%)	2 (33.3%)	0 (0%)
	Kaolack	43	13 (30.2%)	9 (20.9%)	7 (77.8%)
	Kedougou	5	4 (80.0%)	0 (0%)	-
	Kolda	9	9 (100%)	3 (33.3%)	1 (33.3%)

Facility type	Location	Total facilities	Total facilities with >1 facility ownership (% of total facilities)	Total facilities originally with >1 facility type (% of total facilities)	Total facilities originally with >1 facility type and then triangulated or verified (% of total facilities with > 1 facility type)
	Louga	30	12 (40.0%)	9 (30.0%)	1 (11.1%)
	Matam	5	4 (80.0%)	2 (40.0%)	2 (100%)
	Saint-Louis	12	6 (50.0%)	3 (25.0%)	3 (100%)
	Sedhiou	7	4 (57.1%)	3 (42.9%)	1 (33.3%)
	Tambacounda	12	9 (75.0%)	1 (8.3%)	1 (100%)
	Thies	16	11 (68.8%)	3 (18.8%)	1 (33.3%)
	Ziguinchor	10	10 (100%)	2 (20.0%)	2 (100%)
**Health posts**	**Senegal**	**2,349**	1,470 (62.6%)	357 (15.2%)	144 (40.3%)
	Dakar	680	176 (25.9%)	66 (9.7%)	24 (36.4%)
	Diourbel	123	97 (78.9%)	15 (12.2%)	6 (40.0%)
	Fatick	132	113 (85.6%)	23 (17.4%)	13 (56.5%)
	Kaffrine	105	77 (73.3%)	17 (16.2%)	0 (0%)
	Kaolack	151	117 (77.5%)	32 (21.2%)	20 (62.5%)
	Kedougou	48	33 (68.8%)	7 (14.6%)	0 (0%)
	Kolda	101	68 (67.3%)	18 (17.8%)	13 (72.2%)
	Louga	161	118 (73.3%)	16 (9.9%)	4 (25.0%)
	Matam	101	85 (84.2%)	8 (7.9%)	8 (100%)
	Saint-Louis	136	114 (83.8%)	21 (15.4%)	6 (28.6%)
	Sedhiou	75	61 (81.3%)	14 (18.7%)	6 (42.9%)
	Tambacounda	162	127 (78.4%)	76 (46.9%)	19 (25.0%)
	Thies	228	171 (75.0%)	31 (13.6%)	15 (48.4%)
	Ziguinchor	146	113 (77.4%)	13 (8.9%)	10 (76.9%)
**Health huts**	**Senegal**	252 (12.6)	46 (2.3%)	7 (15.2%)	252 (12.6%)
	Dakar	-	-	-	-
	Diourbel	33	0 (0%)	0 (0%)	-
	Fatick	153	4 (2.6%)	1 (0.7%)	0 (0%)
	Kaffrine	155	2 (1.3%)	0 (0%)	-
	Kaolack	172	1 (0.6%)	0 (0%)	-
	Kedougou	85	0 (0%)	1 (1.2%)	0 (0%)
	Kolda	215	3 (1.4%)	8 (3.7%)	6 (75.0%)
	Louga	388	109 (28.1%)	9 (2.3%)	0 (0%)
	Matam	54	0 (0%)	0 (0%)	-
	Saint-Louis	112	18 (16.1%)	19 (17.0%)	0 (0%)
	Sedhiou	119	46 (38.7%)	0 (0%)	-
	Tambacounda	142	62 (43.7%)	5 (3.5%)	0 (0%)
	Thies	268	4 (1.5%)	3 (1.1%)	1 (33.3%)
	Ziguinchor	102	3 (2.9%)	0 (0%)	-

Table S4. Comparing GPS availability and concordance, by facility type, in Senegal and by region. Facilities with more than 1 set of linked GPS coordinates and coordinates varying by more than 2 km could be considered of higher priority for further validation of their geolocation. For a given facility, if multiple GPS coordinates are available and they precisely match, they are not counted as distinct sets of GPS. All facilities here reflect hospitals, health centers, health posts, and health huts (i.e., not facilities designated as "other").

Facility type	Location	Total facilities with GPS	Total facilities with > 1 set of GPS coordinates (% of total facilities with GPS)	Total facilities with > 1 set of GPS and > 2 km of each other (% of total facilities with > 1 set of GPS)
**All facilities**	**Senegal**	**2,423**	1,433 (59.1%)	384 (26.8%)
	Dakar	328	171 (52.1%)	28 (16.4%)
	Diourbel	151	92 (60.9%)	39 (42.4%)
	Fatick	158	105 (66.5%)	25 (23.8%)
	Kaffrine	126	72 (57.1%)	22 (30.6%)
	Kaolack	218	120 (55.0%)	27 (22.5%)
	Kedougou	81	41 (50.6%)	11 (26.8%)
	Kolda	130	67 (51.5%)	19 (28.4%)
	Louga	191	111 (58.1%)	31 (27.9%)
	Matam	128	85 (66.4%)	31 (36.5%)
	Saint-Louis	204	133 (65.2%)	37 (27.8%)
	Sedhiou	102	66 (64.7%)	19 (28.8%)
	Tambacounda	161	92 (57.1%)	26 (28.3%)
	Thies	261	161 (61.7%)	28 (17.4%)
	Ziguinchor	184	117 (63.6%)	41 (35.0%)
**Hospitals**	**Senegal**	**70**	39 (55.7%)	10 (25.6%)
	Dakar	30	13 (43.3%)	2 (15.4%)
	Diourbel	6	4 (66.7%)	0 (0%)
	Fatick	2	2 (100%)	1 (50.0%)
	Kaffrine	2	1 (50.0%)	0 (0%)
	Kaolack	1	1 (100%)	1 (100%)
	Kedougou	1	0 (0%)	-
	Kolda	2	2 (100%)	1 (50.0%)
	Louga	2	2 (100%)	1 (50.0%)
	Matam	3	2 (66.7%)	1 (50.0%)
	Saint-Louis	5	3 (60.0%)	1 (33.3%)
	Sedhiou	2	1 (50.0%)	0 (0%)
	Tambacounda	1	1 (100%)	1 (100%)
	Thies	11	5 (45.5%)	1 (20.0%)
	Ziguinchor	2	2 (100%)	0 (0%)
**Health centers**	**Senegal**	**227**	141 (62.1%)	28 (19.9%)
	Dakar	77	46 (59.7%)	7 (15.2%)
	Diourbel	8	6 (75.0%)	2 (33.3%)
	Fatick	9	8 (88.9%)	0 (0%)
	Kaffrine	4	4 (100%)	2 (50.0%)
	Kaolack	42	9 (21.4%)	1 (11.1%)
	Kedougou	5	4 (80.0%)	1 (25.0%)

Facility type	Location	Total facilities with GPS	Total facilities with > 1 set of GPS coordinates (% of total facilities with GPS)	Total facilities with > 1 set of GPS and > 2 km of each other (% of total facilities with > 1 set of GPS)
	Kolda	7	7 (100%)	1 (14.3%)
	Louga	17	11 (64.7%)	3 (27.3%)
	Matam	5	5 (100%)	1 (20.0%)
	Saint-Louis	11	6 (54.5%)	3 (50.0%)
	Sedhiou	5	5 (100%)	2 (40.0%)
	Tambacounda	12	9 (75.0%)	2 (22.2%)
	Thies	15	13 (86.7%)	2 (15.4%)
	Ziguinchor	10	8 (80.0%)	1 (12.5%)
**Health posts**	**Senegal**	**1,596**	1,185 (74.2%)	320 (27.0%)
	Dakar	221	112 (50.7%)	19 (17.0%)
	Diourbel	109	80 (73.4%)	37 (46.2%)
	Fatick	108	93 (86.1%)	23 (24.7%)
	Kaffrine	85	65 (76.5%)	20 (30.8%)
	Kaolack	132	109 (82.6%)	25 (22.9%)
	Kedougou	39	27 (69.2%)	8 (29.6%)
	Kolda	76	52 (68.4%)	14 (26.9%)
	Louga	126	93 (73.8%)	24 (25.8%)
	Matam	90	70 (77.8%)	24 (34.3%)
	Saint-Louis	125	108 (86.4%)	27 (25.0%)
	Sedhiou	61	50 (82.0%)	13 (26.0%)
	Tambacounda	118	80 (67.8%)	22 (27.5%)
	Thies	179	143 (79.9%)	25 (17.5%)
	Ziguinchor	127	103 (81.1%)	39 (37.9%)
**Health huts**	**Senegal**	**530**	68 (12.8%)	26 (38.2%)
	Dakar	-	-	-
	Diourbel	28	2 (7.1%)	0 (0%)
	Fatick	39	2 (5.1%)	1 (50.0%)
	Kaffrine	35	2 (5.7%)	0 (0%)
	Kaolack	43	1 (2.3%)	0 (0%)
	Kedougou	36	10 (27.8%)	2 (20.0%)
	Kolda	45	6 (13.3%)	3 (50.0%)
	Louga	46	5 (10.9%)	3 (60.0%)
	Matam	30	8 (26.7%)	5 (62.5%)
	Saint-Louis	63	16 (25.4%)	6 (37.5%)
	Sedhiou	34	10 (29.4%)	4 (40.0%)
	Tambacounda	30	2 (6.7%)	1 (50.0%)
	Thies	56	0 (0%)	-
	Ziguinchor	45	2 (7.1%)	0 (0%)

Table S5. Comparing number of facilities in the consolidated facility list with facility numbers reported in previously published facility lists by facility type, by region. SPA 2012-2013 is omitted here as regional counts were not provided in the corresponding report. All facilities here reflect hospitals, health centers, health posts, and health huts (i.e., not facilities designated as "other").

Region	Data source	All facilities	Hospitals	Health centers	Health posts	Health huts
**Dakar**	**Consolidated facility list**	**797**	**35**	**82**	**680**	**0**
	SPA 2017	721	35	39	647	0
	SPA 2019		42	41	664	
	Health Map 2019		16	107	638	
	Health Map 2019 (public only)		15	23	129	
	Health Map 2021 (public only)	204	14	25	126	39
**Diourbel**	**Consolidated facility list**	**173**	**6**	**11**	**123**	**33**
	SPA 2017	150	5	7	97	41
	SPA 2019		5	7	98	
	Health Map 2019		3	19	176	
	Health Map 2019 (public only)		3	9	98	
	Health Map 2021 (public only)	234	4	9	108	113
**Fatick**	**Consolidated facility list**	**296**	**2**	**9**	**132**	**153**
	SPA 2017	261	1	8	104	148
	SPA 2019		1	8	104	
	Health Map 2019		1	17	149	
	Health Map 2019 (public only)		1	7	121	
	Health Map 2021 (public only)	220	1	4	98	117
**Kaffrine**	**Consolidated facility list**	**268**	**2**	**6**	**105**	**155**
	SPA 2017	227	1	4	64	158
	SPA 2019		1	5	64	
	Health Map 2019		1	8	100	
	Health Map 2019 (public only)		1	4	94	
	Health Map 2021 (public only)	375	1	4	116	254
**Kaolack**	**Consolidated facility list**	**370**	**4**	**43**	**151**	**172**
	SPA 2017	296	4	6	97	189
	SPA 2019		8	6	98	
	Health Map 2019		1	13	183	
	Health Map 2019 (public only)		1	4	113	
	Health Map 2021 (public only)	375	1	4	116	254
**Kedougou**	**Consolidated facility list**	**139**	**1**	**5**	**48**	**85**
	SPA 2017	101	0	3	28	70
	SPA 2019		0	4	29	
	Health Map 2019			6	47	
	Health Map 2019 (public only)			3	39	
	Health Map 2021 (public only)	130	1	4	42	83
**Kolda**	**Consolidated facility list**	**327**	**2**	**9**	**101**	**215**
	SPA 2017	349	2	8	75	264
	SPA 2019		2	10	76	
	Health Map 2019		1	9	95	

Region	Data source	All facilities	Hospitals	Health centers	Health posts	Health huts
	Health Map 2019 (public only)		1	4	69	
	Health Map 2021 (public only)	325	1	4	76	244
**Louga**	**Consolidated facility list**	**582**	**3**	**30**	**161**	**388**
	SPA 2017	272	3	14	129	126
	SPA 2019		3	14	133	
	Health Map 2019		2	9	155	
	Health Map 2019 (public only)		2	4	116	
	Health Map 2021 (public only)	480	2	10	118	350
**Matam**	**Consolidated facility list**	**163**	**3**	**5**	**101**	**54**
	SPA 2017	136	1	5	76	54
	SPA 2019		1	5	76	
	Health Map 2019		2	9	106	
	Health Map 2019 (public only)		2	4	96	
	Health Map 2021 (public only)	186	3	9	102	72
**Saint-Louis**	**Consolidated facility list**	**265**	**5**	**12**	**136**	**112**
	SPA 2017	214	2	6	111	95
	SPA 2019		2	6	111	
	Health Map 2019		3	21	154	
	Health Map 2019 (public only)		3	8	112	
	Health Map 2021 (public only)	321	3	9	123	186
**Sedhiou**	**Consolidated facility list**	**204**	**3**	**7**	**75**	**119**
	SPA 2017	118	1	4	43	70
	SPA 2019		1	4	43	
	Health Map 2019		1	6	72	
	Health Map 2019 (public only)		1	3	62	
	Health Map 2021 (public only)	167	1	4	60	102
**Tambacounda**	**Consolidated facility list**	**317**	**1**	**12**	**162**	**142**
	SPA 2017	193	1	18	79	95
	SPA 2019		1	17	59	
	Health Map 2019		1	20	155	
	Health Map 2019 (public only)		1	7	125	
	Health Map 2021 (public only)	284	1	7	148	128
**Thies**	**Consolidated facility list**	**524**	**12**	**16**	**228**	**268**
	SPA 2017	486	10	14	177	285
	SPA 2019		11	14	178	
	Health Map 2019		5	24	349	
	Health Map 2019 (public only)		3	9	180	
	Health Map 2021 (public only)	492	5	10	180	297
**Zinguichor**	**Consolidated facility list**	**260**	**2**	**10**	**146**	**102**
	SPA 2017	240	2	12	126	100
	SPA 2019		2	12	126	
	Health Map 2019		2	14	184	
	Health Map 2019 (public only)		2	5	124	
	Health Map 2021 (public only)	237	2	5	114	116

Table S6. Comparing number of facilities with GPS in the consolidated facility list with facility numbers from Maina and colleagues, nationally and by region. All facilities here reflect hospitals, health centers, health posts, and health huts (i.e., not facilities designated as "other").

Region	Data source	All facilities	Hospitals	Health centers	Health posts	Health huts
**Senegal**	**Consolidated facility list**	**2,423**	**70**	**227**	**1,596**	**530**
	Maina et al 2019	1,256	29	83	1,144	
**Dakar**	**Consolidated facility list**	**328**	**30**	**77**	**221**	
	Maina et al 2019	121	10	14	97	
**Diourbel**	**Consolidated facility list**	**151**	**6**	**8**	**109**	**28**
	Maina et al 2019	91	3	6	82	
**Fatick**	**Consolidated facility list**	**158**	**2**	**9**	**108**	**39**
	Maina et al 2019	99	1	6	92	
**Kaffrine**	**Consolidated facility list**	**126**	**2**	**4**	**85**	**35**
	Maina et al 2019	71	1	4	66	
**Kaolack**	**Consolidated facility list**	**218**	**1**	**42**	**132**	**43**
	Maina et al 2019	77	1	2	74	
**Kedougou**	**Consolidated facility list**	**81**	**1**	**5**	**39**	**36**
	Maina et al 2019	30		3	27	
**Kolda**	**Consolidated facility list**	**130**	**2**	**7**	**76**	**45**
	Maina et al 2019	53	1	3	49	
**Louga**	**Consolidated facility list**	**191**	**2**	**17**	**126**	**46**
	Maina et al 2019	113	2	12	99	
**Matam**	**Consolidated facility list**	**128**	**3**	**5**	**90**	**30**
	Maina et al 2019	79	2	4	73	
**Saint-Louis**	**Consolidated facility list**	**204**	**5**	**11**	**125**	**63**
	Maina et al 2019	119	2	4	113	
**Sedhiou**	**Consolidated facility list**	**102**	**2**	**5**	**61**	**34**
	Maina et al 2019	49	1	2	46	
**Tambacounda**	**Consolidated facility list**	**161**	**1**	**12**	**118**	**30**
	Maina et al 2019	88	1	7	80	
**Thies**	**Consolidated facility list**	**261**	**11**	**15**	**179**	**56**
	Maina et al 2019	156	2	11	143	
**Zinguichor**	**Consolidated facility list**	**184**	**2**	**10**	**127**	**45**
	Maina et al 2019	110	2	5	103	

Supplementary file 1. Report from the facility list workshop in Dakar, Senegal from January 31-February 1, 2023 (available in French only).

MINISTÈRE DE LA SANTÉ ET DE L'ACTION SOCIALE

IRESSEF

Gates Ventures

EXEMPLARS IN GLOBAL HEALTH

RAPPORT

ATELIER DE PARTAGE SUR LA CARTOGRAPHIE DES ETABLISSEMENTS DE SANTE AU SENEGAL (MAPPING HEALTH FACILITIES LIST)

Table des matières

1. CONTEXTE ET JUSTIFICATION 3
2. OBJECTIFS DE L'ATELIER 4
3. DEROULEMENT 4
4. PROCHAINES ETAPES ET RECOMMANDATIONS : 7

1. CONTEXTE ET JUSTIFICATION

La révolution numérique offre des perspectives prometteuses en matière d'amélioration de la gestion des données sanitaires de routine dans les pays en développement. Cependant, Cet élan est parfois brisé par le manque d'engagement politique, le manque de coordination des efforts et la mauvaise planification du système d'information sanitaire (SIS). Cela peut entrainer des échecs, la non-rationalisation des ressources et conduire, par ailleurs, à l'abandon ou aux contreperformances du SIS.

Au cours de la dernière décennie, plusieurs systèmes d'information sanitaire de routine (SISR) des pays en développement ont été transférés sur des plateformes électroniques. Cependant, les évaluations de ces systèmes électroniques ont donné lieu à des résultats mitigés ; par exemple, certains résultats ont fourni des preuves d'un système prometteur, tandis que d'autres ont suggéré l'échec du système. L'adoption et la migration ultérieure des systèmes sur papier vers des plates-formes électroniques présenteront toutefois de nouveaux défis. Par exemple, une évaluation faite au Sénégal a montré quelques faiblesses, parmi lesquelles il est à noter l'inexistence de plan directeur pour articuler au mieux les stratégies de développement de l'information sanitaire ; la multiplicité des sous-systèmes d'information sanitaire et l'absence de coordination effective du système statistique national. Il est donc nécessaire d'établir des processus qui garantissent le maintien et la promotion de l'intégrité et de l'évolutivité du système. Un effort coordonné doit intégrer une approche systémique, une vue d'ensemble et une planification à grande échelle, ce qui inclut la sélection et l'intégration de normes dans le développement du système d'information électronique, comme l'a demandé l'Organisation mondiale de la santé dans la résolution de la 66e assemblée sur la "santé en ligne". L'une des normes requises est un processus d'identification unique des établissements de santé qui entreront dans le système d'information. Les processus d'identification peuvent être complexes, notamment au Sénégal où il existe plusieurs programmes et sous-systèmes qui ne répondent pas aux mêmes objectifs et qui sont gérés par différentes directions. Si ces efforts ne sont pas coordonnés et que le processus d'identification des établissements de santé ne sont pas bien ficelés, la viabilité du SIS et des objectifs attendus pourraient être compromis.

C'est dans ce contexte que la DPRS et le COUS en collaboration avec l'IRESSEF ont travaillé entre Mars et Aout 2022, avec les partenaires de la Gates-Ventures pour la mise sur pieds d'une liste principale des établissements de santé (MFL) qui intègre les types, noms et coordonnées géographiques de l'ensemble des établissements de santé au Sénégal. Ce travail a consisté à trianguler plusieurs sources de données pour obtenir une liste complète des établissements de santé. Au sortir de ce processus, il est convenu d'organiser un atelier de partage de deux jours avant la fin de l'année pour partager les résultats obtenus avec quelques acteurs de la santé du niveau central et du niveau régional. Il s'agira ensuite de fixer ensemble les prochaines étapes et définir le dispositif de coordination et de suivi des activités.

2. OBJECTIFS DE L'ATELIER

Les objectifs de la rencontre sont de :

- Présenter la liste principale des établissements de santé (MFL) conçue ;
- Initier des discussions sur l'amélioration, l'optimisation et la capitalisation de la liste ;
- Définir le mécanisme de coordination et de suivi à mettre en place **;** et
- Définir les perspectives quant à l'utilisation de la liste par les programmes.

3. DEROULEMENT DE LA SESSION

Les activités de la première journée de « l'atelier de partage de la cartographie nationale des établissements de santé » ont démarré à 09H50MN avec des mots de bienvenue du Directeur du Centre des Opérations d'Urgence Sanitaire (COUS) à l'endroit de ses invités. L'atelier, tenu en bimodal à la salle de conférence du COUS, a vu la participation de plusieurs entités notamment les services du Ministère de la Santé et de l'Action sociale (MSAS) tels que la Direction de la Santé de la Mère et de l'Enfant(DSME), la Direction des Etablissements Privés de Santé(DEPS), la Direction de la Planification, de la Recherche et des Statistiques (DPRS), les régions médicales, les partenaires tels que IRESSEF, Gates Ventures, PATH, Bluesquare, Health Site, entre autres.

Le président de séance, en l'occurrence le Directeur du COUS s'est félicité de la tenue d'un atelier de cette nature avec les différents profils qui sont conviés à la rencontre.

Après les discours de bienvenue, un tour de table s'en est suivi pour la présentation des différents participants. Au terme de ceci, un facilitateur de l'IRESSEF a procédé à la présentation de l'agenda. A la suite de ce dernier, le représentant de l'OSM (Openstreetmap) a tenu à partager brièvement les travaux de cartographie des établissements de santé qu'ils ont réalisé dans les régions de saint Louis et de Matam. En d'autres termes, L'OSM a réalisé une phase pilote dans la région Saint Louis et s'est récemment déployé au niveau de la région de Matam ou il a pu réaliser la cartographie complète des établissements de santé.Suite à ce bref rappel, une fenêtre de discussion a été ouverte pour permettre aux participants de donner leurs impressions sur ce travail. Les points de discussions ont porté principalement sur : le déroulement de la campagne de collecte d'information à Saint-Louis ; et les éléments recherchés lors de cette campagne.

Pour répondre à ces interpellations, le représentant de l'OSM a affirmé que l'étude a été faite pour l'ensemble des structures de santé de la région partant de la case à l'hôpital et que toutes les informations à propos des horaires de travail de ces structures sont disponibles de même que leurs géolocalisations complètes. Des suggestions pour parfaire cette production sont ressorties lors des échanges et il se trouve que PATH est en train d'effectuer le même travail dans la zone Nord. C'est ainsi qu'il leur a été recommandé de mutualiser leurs efforts pour être beaucoup plus efficient. A ce sujet, le COUS suggère aux différentes régions d'organiser des réunions d'harmonisation des interventions de ces organismes.

Au terme de ces échanges, une pause-café a été observée à 10H38MN. A la reprise à 11H13MN, l'IRESSEF a déroulé une première présentation sur le développement d'une liste unifiée et géo localisée des établissements de santé au Sénégal. C'est ainsi que le facilitateur est revenu sur les grandes lignes de ce projet. Selon lui, l'idée principale était de disposer de données complètes et régulièrement mises à jour sur le nombre et le type d'établissement de santé existant qui pourront servir lors de la planification des programmes. Ce projet arrive dans un contexte où le Sénégal n'avait à ce jour aucune « Master Facility List » centralisée avec des informations de géolocalisation même si certains supports comme la Carte Sanitaire étaient disponibles.

Au décours de ce chapitre sur la justification de ce projet, un point sur ce qu'est la MFL a été abordé afin de permettre aux différents participants d'être au même niveau d'information. En résumé, pour comprendre ce qu'elle est réellement, il faut noter que la MFL est une liste complète des établissements de santé que les initiateurs ont l'intention de mettre à jour tous les deux ans. Le facilitateur est également revenu sur le travail abattu depuis le démarrage de ce projet jusqu'à aujourd'hui. Afin d'aboutir sur ces résultats partagés lors de cet atelier, le processus utilisé consistait à trianguler des données de différentes sources (ANSD, MSAS, etc.) jusqu'à obtenir une base consolidée et géo localisée. Quelques applications de cet outil ont été présentées aux participations en guise d'exemple.

Concernant l'état d'avancement des travaux, pour tous les établissements confondus, 55,3% des établissements présents sur la liste sont géo localisée. Le facilitateur a aussi passé en revue les difficultés rencontrées dans ce projet telles que l'inexistence d'une base unifiée qui pourrait servir de référence, l'identification des établissements opérationnels à l'heure actuelle, entre autres.

Les priorités du moment restent la confirmation et la validation des listes avec les différentes parties prenantes. A ce sujet, une collaboration avec le MSAS permettra de poursuivre au mieux les efforts fournis jusque-là.

Au terme de cette présentation, une plage de discussion fut ouverte. Les interventions portaient essentiellement sur :

- La liste des postes qui ne figurent pas sur la liste MFL ;
- L'accessibilité de la liste une fois qu'elle sera corrigée ;
- Les outils utilisés pour le recensement des données de la présente liste ;
- La liste exhaustive des structures privées fonctionnelles ;
- Les doublons présents sur la liste ;
- Les sources manquantes dans la collecte des données ;
- La dénomination correcte des établissements de santé ;
- Etc.

Pour réagir par rapport aux interpellations, l'IRESSEF affirme que son souhait est d'avoir à la fin une liste en ligne interactive conçue avec des normes d'utilisation. Quant aux sources, il rappelle que les chercheurs ont exploité des bases de données déjà disponibles. Il souligne qu'il faut également le concours de la direction des établissements privés pour le recensement complet de ses structures. Comme contribution, la DGES s'est engagée à fournir à l'IRESSEF une liste complète des hôpitaux avec leurs noms officiels de même que le nombre exact de structures de santé au Sénégal.

Au terme de ces interactions fructueuses, le président de séance s'est félicité de la participation active de tous. Il soutient que les recommandations sorties de cette rencontre pourront faciliter les prochaines étapes dans la collecte des données pour la MFL.

L'objectif de la journée étant atteint, la séance fut levée.

A la deuxième journée de l'atelier, les travaux ont commencé à partir de 10h avec la lecture du rapport de la première journee. Après le partage de ce document, le débat fut ouvert afin de donner l'occasion aux participants d'apporter leurs observations. C'est ainsi que quelques suggestions ont été fournies pour parfaire le rapport. A la suite de ces échanges, le rapport fut adopté sous réserve d'intégration des remarques émises par l'assistance.
Les travaux de la journée se poursuivirent avec une présentation de BlueSquare portant sur les géo données et la partage des termes de référence du Groupe de Travail qui constituera le comité de suivi des travaux sur la MFL. Ce groupe de travail s'activera autour de la création d'un Master Facility List. L'idée de la mise sur pieds d'une telle instance est la mise en place d'une interface web qui va regrouper les données de tous ceux qui travaillent sur les géo données au Sénégal (CSSDOS, COUS, DPRS, IRESSEF BlueSquare, Healthsites, Path, etc.). Ainsi, le facilitateur affirme que l'ébauche de ce projet de terme de référence a été soumis à la CSSDOS pour approbation. Dans ces TDR on peut voir les points saillants ci-dessous :

- La mission du Groupe de Travail (GT) ;
- Le rôle du GT ;
- La composition de ce groupe ;
- Les membres du GT (au niveau national et international) ;
- La méthodologie qui sera utilisée lors des travaux ;
- La feuille de route du GT et les prochaines étapes jusqu'aux démarrages des travaux.

Ce groupe aura donc comme tâche principale l'harmonisation des interventions concernant les données à caractères géo spatial et la répartition des rôles pour la mise à jour des données. Le GT devrait également s'assurer que la CSSDOS dispose de tous les outils en tant que responsable de la MFL et du futur géo-registre. Parmi les missions qui lui sont assignées, notons que cette instance devra apporter une assistance technique pour l'amélioration des bases de données et le renforcement des capacités des différents membres qui participeront à ce processus. Bluesquare propose dans les TDR, de mettre à la tête du GT la CSSDOS et comme rapporteur l'ANAT (Agence Nationale de l'Aménagement du Territoire).

Comme étapes suivantes dans ce processus de création du GT, on a :

- La validation des termes de référence ;
- La signature de la note par le SG ;
- Et enfin une rencontre en fin février pour rendre compte sur l'état d'avancement des activités d'installation de cette instance.

A la suite de la présentation de la proposition de TDR, la parole fut donnée aux participants. Au cours des débats, des suggestions sont ressorties, pour l'essentiel on peut retenir :

- L'amélioration de la composition du GT : les membres de l'atelier de partage sont intégrés dans l'équipe ainsi que d'autres profils sélectionnés par les participants ;
- L'implication effective de la CSSDOS, qui a un rôle pivot dans ce GT ;
- La révision des aspects techniques des travaux prévus ;
- L'harmonisation des outils de collectes ;
- La tenue d'une date pour un partage des bilans d'étape ;
- L'utilisation des données du DHIS2.

Au terme de ces échanges, les activités furent suspendues pour une pause-café à 11H25MN. A la reprise à 12H.

4. PROCHAINES ETAPES ET RECOMMANDATIONS :

Au cours des échanges faits, plusieurs recommandations et perspectives ont été relevés dont les plus pertinentes sont listées ci-dessous :

- L'implication des acteurs du niveau opérationnel dans le processus des collectes de données et la formation de ces derniers ;
- La mise en place d'un comité de suivi piloté par le MSAS pour assurer le suivi ;
- L'utilisation des bases de données de la CSSDOS (rapport de la carte sanitaire 2022), de Healthsite de Bluesquare et de PATH ;
- L'utilisation de la liste de dénomination de la DGES ;
- Le recours à la direction des établissements privés pour avoir un aperçu sur les structures privées fonctionnelles et en règle ;
- La sécurité des données lors des partages.
- La tenue d'une réunion préparatoire de la première rencontre du GT (avec un point autour de la nomenclature des structures et les procédures de partage des données (la semaine du 13 a été proposée));
- La tenue de la réunion du GT (les semaines du 20 où du 27 sont proposées) ;
- La signature de la note par le SG du MSAS (à suivre jusqu'à sa réalisation) ;
- Le lancement des activités du GT.

A l'issue des travaux, le président de séance a dressé un bilan satisfaisant de ces deux journées de partage sans oublier de saluer l'assiduité des participants durant ces deux jours et leurs contributions enrichissantes aux travaux. Il a aussi insisté sur l'application des recommandations fournies pour atteindre les objectifs fixés par le groupe avant de déclarer clos l'atelier.
